# Pulmonary Vascular Permeability and Extravascular Lung Water Index Vary with Infection Site in Patients with Liver Cirrhosis and Septic Shock

**DOI:** 10.3390/jcm15166412

**Published:** 2026-08-19

**Authors:** Hsiang-Ting Keng, Ming-Ling Chang, Rong-Nan Chien, Yung-Chang Chen, Ya-Chung Tian, Yun-Shing Peng, Hui-Chun Huang, Ji-Tseng Fang, Fa-Yauh Lee, Ming-Hung Tsai

**Affiliations:** 1 Division of Gastroenterology and Hepatology, Chang Gung Memorial Hospital, Linkou Medical Center, Chang Gung University, Taoyuan 333, Taiwan; keng1227@cgmh.org.tw (H.-T.K.); mlchang8210@gmail.com (M.-L.C.); ronald@cgmh.org.tw (R.-N.C.); 2 Division of Critical Care Nephrology, Kidney Institute, Chang Gung Memorial Hospital, Linkou Medical Center, Chang Gung University, Taoyuan 333, Taiwan; cyc2356@gmail.com (Y.-C.C.); dryctian@cgmh.org.tw (Y.-C.T.);; 3 Department of Internal Medicine, Chang Gung Memorial Hospital, Chia-Yi 613, Taiwan; pengys66@gmail.com; 4 Division of Gastroenterology and Hepatology, Department of Medicine, Taipei Veteran General Hospital, Faculty of Medicine, Yang-Ming University School of Medicine, Taipei 112, Taiwan; fylee@vghtpe.gov.tw; 5 Division of General Medicine, Department of Medicine, Taipei Veteran General Hospital, Faculty of Medicine, Yang-Ming University School of Medicine, Taipei 112, Taiwan

**Keywords:** cirrhosis, non-SBP sepsis, lung edema, pulmonary vascular permeability index, extravascular lung water index

## Abstract

**Background:** Cirrhotic patients with septic shock have variable responses to albumin infusion depending on the infection site. While albumin improves outcomes in spontaneous bacterial peritonitis (SBP), it offers no benefit in non-SBP infections and increases the risk of pulmonary edema, particularly in cirrhotic patients with pneumonia. We hypothesized cirrhotic patients with septic shock due to pneumonia would exhibit higher pulmonary vascular permeability index (PVPI) values. Using Pulse index Continuous Cardiac Output (PiCCO) monitoring, we examined the impact of infection site on PVPI and extravascular lung water index (EVLWI). **Methods:** We conducted a retrospective cohort study of 147 cirrhotic patients with septic shock. All patients underwent PiCCO monitoring. Measurements including PVPI, EVLWI, disease severity scores, and laboratory biomarkers on the first and second day of ICU admission were analyzed. **Results:** Pneumonia was the most common infection and was associated with significantly elevated PVPI and EVLWI values on both day 1 and day 2 compared to the non-pneumonia group. By contrast, patients with SBP had significantly lower PVPI and EVLWI values than those with non-SBP infections. Within the pneumonia subgroup, 28-day non-survivors had higher PVPI and EVLWI values than survivors. PVPI on day 2 was the strongest predictor of 28-day mortality (AUROC 0.83) among the PiCCO parameters. A PVPI on day 2 > 2.65 identified a high-risk phenotype, with a higher 28-day mortality (77.1% vs 18.9%, *p* < 0.001). PVPI on day 2 remained an independent predictor of 28-day mortality after adjustment for disease severity. Additionally, PaO_2_/FiO_2_ ratio on day 2 was an independent factor associated with day-2 PVPI ≤ 2.65. **Conclusions:** In cirrhotic patients with septic shock, non-SBP infections, mostly from pneumonia, were associated with elevated PVPI and EVLWI values. PVPI-based risk stratification may help optimize resuscitation strategies for patients at risk of pulmonary edema while reducing heterogeneity in future clinical trials.

## 1. Introduction

Patients with cirrhosis are susceptible to sepsis and septic shock [1,2]. Albumin is currently recommended for several established indications, including the prevention of post-paracentesis circulatory dysfunction after large-volume paracentesis, treatment of hepatorenal syndrome, and spontaneous bacterial peritonitis (SBP) [2,3,4]. In regards to SBP, intravenous albumin has represented a major therapeutic advance. However, the benefits of albumin administration in cirrhotic patients with non-SBP infections remain uncertain [3,4,5,6,7]. Safety concerns have been raised regarding the development of pulmonary edema following albumin infusion in patients with non-SBP infections, particularly among those with pneumonia [3,4,5,6,7], which may attenuate or even offset the potential benefits of treatment in this specific subgroup. In a multicenter randomized controlled trial, albumin infusion in addition to standard care for cirrhotic patients with non-SBP infections did not improve the survival rate, and pulmonary edema was reported in 8.3% of patients receiving albumin, with pneumonia accounting for half of these cases [5]. Indeed, two clinical trials [4,6,7] had to be prematurely terminated due to a lack of efficacy and a higher incidence of pulmonary edema associated with albumin administration. Likewise, in another randomized controlled study, the ALPS trial, resuscitation for patients with cirrhosis and septic shock using 20% albumin conferred no survival benefit over balanced crystalloid (plasmalyte) and was also associated with a higher rate of pulmonary complications (44% vs 20%) [8]. Moreover, the presence of pneumonia independently increased the odds of developing pulmonary complications by around five-fold (adjusted OR 5.26, 95% CI 1.56–17.78) [8]. Although these findings have raised doubts about the use of albumin as a “one-size-fits-all” strategy for the management of cirrhotic patients with sepsis, the mechanisms underlying these discrepant outcomes remain poorly understood.

Liver cirrhosis with portal hypertension is characterized by the up-regulation of endothelial nitric-oxide synthase (eNOS) and excess nitric oxide (NO) production, which drives widespread vasodilatation, including the pulmonary vasculature [9]. Moreover, the eNOS-NO pathway serves as a critical mediator of vascular permeability regulated by the vascular endothelial growth factor (VEGF) [10,11] and likely contributes to the heightened pulmonary vascular permeability reported in cirrhotic patients [9,12]. Studies have shown that pneumonia triggers diffuse alveolar damage with neutrophilic inflammation and cytokine release, disrupting the alveolar–capillary barrier, thus potentially increasing pulmonary permeability [13,14]. Therefore, we hypothesized that cirrhotic patients with septic shock from pneumonia would exhibit even greater pulmonary permeability than those with other infections, which might explain their greater propensity for pulmonary edema during albumin infusion observed in the previous clinical trials involving patients with non-SBP infections [5,6,7].

The Pulse indicator Continuous Cardiac Output (PiCCO) monitoring device provides a quantitative assessment of pulmonary permeability and extravascular lung water. It is a widely validated hemodynamic monitoring tool for shock patients in the intensive-care unit. Using thermodilution curve analysis, it quantifies important measurements for hemodynamic monitoring, including the cardiac output, preload, systemic vascular resistance, extravascular lung water index (EVLWI), and pulmonary vascular permeability index (PVPI) [15,16]. EVLWI reflects the amount of fluid accumulated in the interstitial and alveolar space, whereas PVPI serves as a surrogate for alveolar–capillary barrier integrity and pulmonary permeability [17]. Elevations in both indices are hallmarks of acute lung injury and are associated with unfavorable outcomes in patients with septic shock, irrespective of the presence of underlying cirrhosis [18,19,20]. 

To test our hypothesis, we conducted this retrospective study comparing PVPI and EVLWI across different infection sites in cirrhotic patients with septic shock using PiCCO monitoring, with particular emphasis on pneumonia.

## 2. Method

### 2.1. Study Design and Patient Eligibility

This study was conducted with approval from the institutional review board of Chang Gung Memorial Hospital (IRB202400261B0, issued on 5 Mar 2024) and conducted in accordance with the Declaration of Helsinki of the World Medical Association. Patient consent was waived due to the retrospective nature of this study. This study is a retrospective analysis of prospectively collected data from critically ill patients with liver cirrhosis admitted to the ICU. Adult patients with liver cirrhosis and septic shock admitted to a specific gastroenterology ICU at Chang Gung Memorial Hospital, a 3700-bed medical center in Linkou, Taiwan, between Jan 2017 and Mar 2020 were included. The attending physician in charge is dual board-certified in gastroenterology and critical care medicine. Cirrhosis was diagnosed histologically or based on clinical imaging and laboratory findings. Liver cirrhosis was clinically diagnosed in patients with esophageal varices or ascites without an alternative cause and with unequivocal cirrhotic findings on ultrasonography or computed tomography. Septic shock was defined according to the Sepsis-3 criteria [21]. Patients with pre-existing heart failure or concurrent hypovolemia and those who died on the first day of ICU admission were excluded. All patients were resuscitated according to the standardized treatment protocol for septic shock [22]. In addition, albumin infusion (25 g/day for 2 consecutive days) was routinely administered as part of the standardized resuscitation strategy for septic shock under Taiwan’s National Health Insurance reimbursement policy. The primary infection site of septic shock on the first day of ICU admission was documented as the infection focus in our study, based on a comprehensive review of medical records, chest radiography, laboratory data, and clinical microscopic or microbiological studies from potential sources, such as sputum, urine, ascites, and blood.

### 2.2. Measurements

Measurements on day 1 and day 2 of ICU admission were collected in our analysis. Upon admission, laboratory tests including hematologic and biochemical panels were performed routinely for all patients. Clinical microscopic or microbiological studies from potential sources, such as sputum, urine, ascites, and blood, were also obtained if available. Disease severity was assessed with the Model for End-Stage Liver Disease (MELD), Acute Physiology and Chronic Health Evaluation II (APACHE II), Sequential Organ Failure Assessment (SOFA), and the Child–Pugh scores [23].

Under Taiwan’s National Health Insurance program, PiCCO monitoring is reimbursed for all patients with shock. In this cohort, the decision to place a PiCCO catheter was made at the discretion of the physician. PiCCO measurements were taken by trained ICU personnel according to the manufacturer’s recommendations. The hemodynamic measurements were calculated using the PiCCO monitoring device (Getinge/PULSION Medical system, Feldkirchen, Germany), which employs a single thermal indicator technique. Initially, 15 to 20 mL of iced (<6 °C) 0.9% saline was injected via a central venous catheter into superior vena cava, and the temperature change was detected by a thermistor-tipped femoral arterial catheter. Cardiac output (CO) was calculated from the thermodilution curve using the Stewart–Hamilton method. Each measurement was obtained in triplicate and averaged. Measurements were repeated if the variability between cardiac output measurements exceeded 10%. This method has been validated, and its performance is comparable with that of pulmonary artery thermodilution [17]. Volumetric variables were derived from CO, the mean transit time of the thermal indicator, and the decay time of the thermodilution curve. Briefly, volumetric variables were calculated using standard transpulmonary thermodilution algorithms implemented in the PiCCO system. Intrathoracic thermal volume (ITTV) was calculated as cardiac output (CO) × mean transit time (MTt), whereas pulmonary thermal volume (PTV) was calculated as CO × exponential downslope time (DSt). Global end-diastolic volume (GEDV) was calculated as ITTV − PTV, and intrathoracic blood volume (ITBV) was calculated as 1.25 × GEDV. Pulmonary blood volume (PBV) was calculated as ITBV − GEDV. Extravascular lung water (EVLW) was calculated as ITTV – ITBV. The pulmonary vascular permeability index (PVPI) was computed as EVLWI divided by pulmonary blood volume.

All volumetric and hemodynamic variables were indexed to body surface area, except extravascular lung water (EVLW). EVLW was indexed to the following: (1) actual body weight (aEVLWI), calculated as EVLW divided by the patient’s daily weight (kg); or (2) predicted body weight (pEVLWI), calculated as EVLW divided by predicted body weight (kg) using the Devine formula: 0.91 [height (cm) − 152.4] + 50 for males or +45.5 for females [24]. aEVLWI represents the conventional PiCCO-derived index. Because extravascular lung water is more closely related to lung size than body mass, aEVLWI may underestimate pulmonary edema in obese patients and overestimate it in underweight patients. In contrast, pEVLWI is indexed to predicted body weight, which better approximates lung volume and is therefore considered more physiologically appropriate, particularly in patients with extreme body habitus [24]. 

### 2.3. Statistical Analysis

Descriptive statistics are reported as mean ± SD or median with interquartile range (IQR) in parentheses. Normality of continuous variables was assessed with the Kolmogorov–Smirnov test. Student’s t test was used for normally distributed continuous variables; otherwise, the Mann–Whitney U test was used. Categorical variables were compared with the Chi-square (χ^2^) test. Discriminative performance of the PiCCO parameters for 28-day mortality in pneumonia patients was tested by the area under the receiver operating characteristic curve (AUROC). ROC analysis was performed to determine the optimal cut-off by Youden index (sensitivity + specificity − 1). Kaplan–Meier curve was used for survival analysis. Meanwhile, risk factors of 28-day mortality and high pulmonary permeability among pneumonia patients were assessed using univariate and multivariate analyses by logistic regression to obtain independent risk factors. Risk factors were first evaluated by univariate logistic regression analysis. Variables with a *p* value <0.10 in the univariate analysis were selected for the multivariable logistic regression model. Variable selection was performed using the Forward Likelihood Ratio (Forward LR) method. Variables that remained statistically significant (*p* <0.05) were retained in the final model. The events-per-variable (EPV) ratio was calculated for the multivariable logistic regression model. Furthermore, to assess the internal validity and stability of the optimal cut-off value, a bootstrap internal validation procedure was performed using 1000 resamples with replacement from the original cohort. The correlation between the PaO_2_/FiO_2_ and the PVPI was analyzed using the Spearman method. All tests were two-sided with a significance level set at *p* < 0.05. Primary analyses were performed in SPSS version 20 for Windows (IBM, Armonk, NY, USA). Bootstrap internal validation and ROC cut-off stability analyses were performed using R version 4.2.3 (R Foundation for Statistical Computing, Vienna, Austria).

## 3. Results

**Figure 1:**
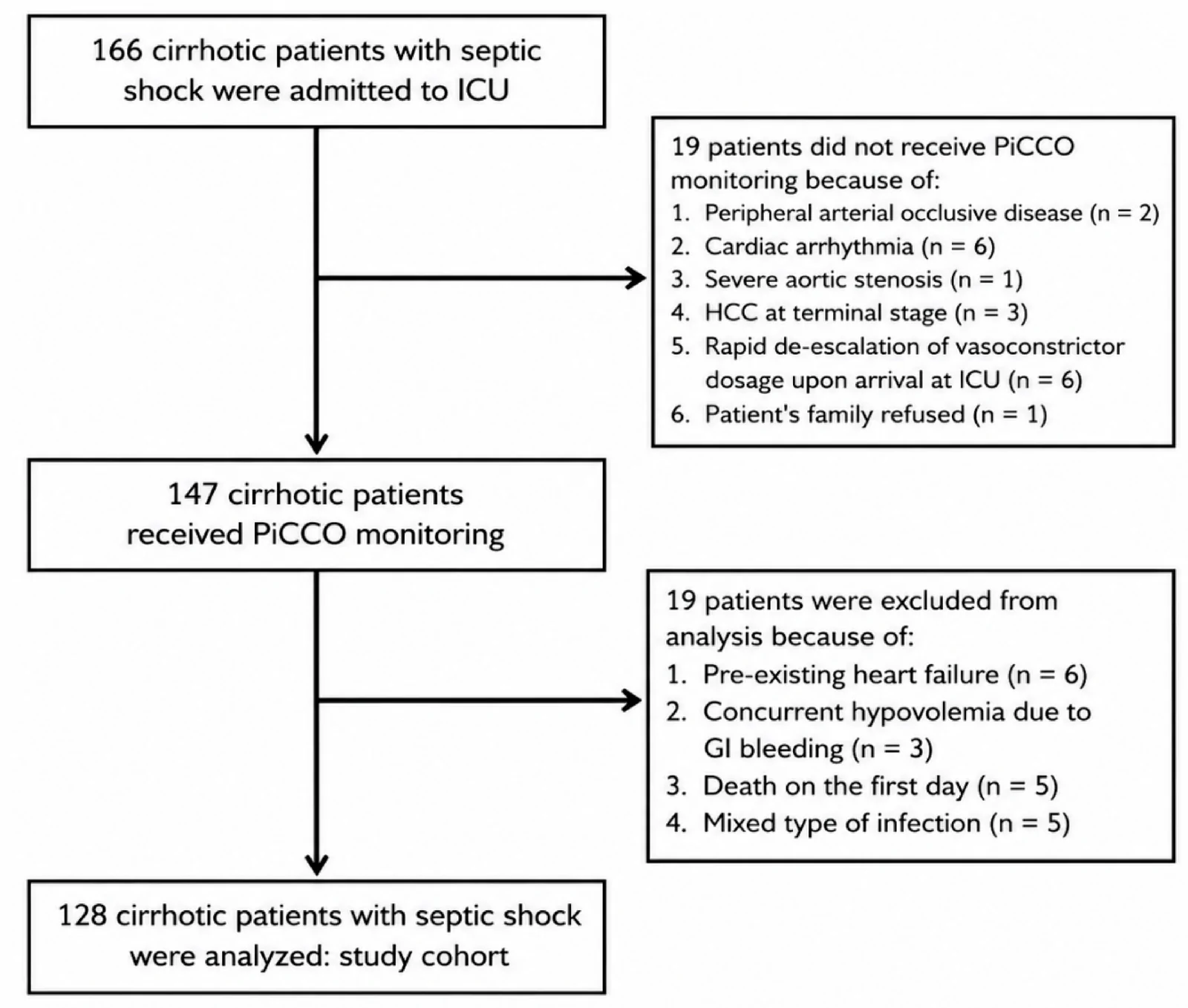
Diagram showing the flow of patients included in the study cohort.

During the study period, 166 cirrhotic patients with septic shock were admitted to the ICU. Among them, 147 underwent PiCCO monitoring. Reasons for not receiving PiCCO were peripheral arterial occlusive disease (n = 2), cardiac arrhythmia (n = 6), severe aortic stenosis (n = 1), terminal-stage HCC (n = 3), rapid de-escalation of vasoconstrictor dosage upon ICU arrival (n = 6), and family refusal (n = 1). Among the 147 patients with PiCCO monitoring, 19 were excluded because of pre-existing heart failure (n = 6), concurrent hypovolemia due to GI bleeding (n = 3), death on the first day (n = 5), or a mixed type of infection (n = 5), leaving 128 patients in the final analysis. Figure 1 shows the flow of patients included in the study cohort. 

**Figure 2:**
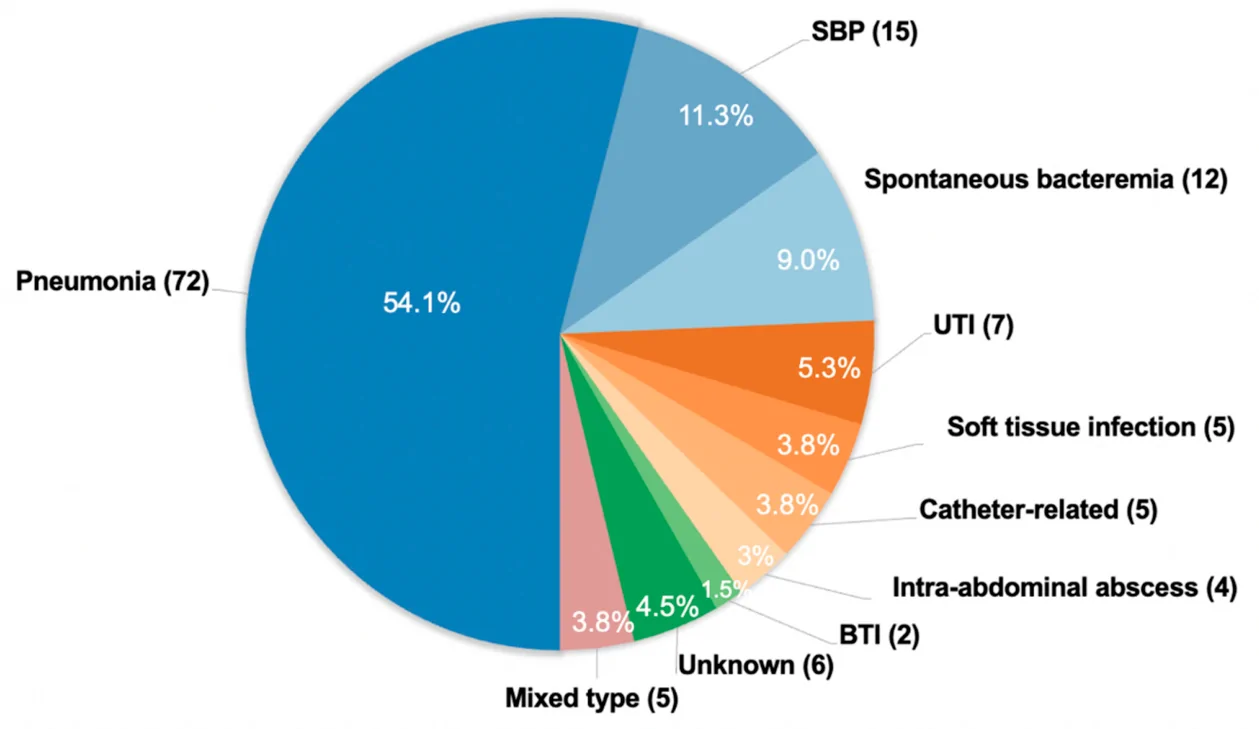
Distribution of infection sites in cirrhotic patients with septic shock (n = 133).

Pneumonia was the most common infection focus (n = 72), followed by SBP (n = 15) and spontaneous bacteremia (n = 12) (Figure 2). Patients with SBP showed significantly lower PVPI and EVLWI values than those with non-SBP infections on both day 1 and day 2 of ICU admission (Table 1). Conversely, patients with pneumonia had significantly higher PVPI and EVLWI values than those with non-pneumonia infections on both days. Moreover, pneumonia patients had greater disease severity, indicated by higher APACHE II scores on day 1 and day 2 and higher SOFA scores on day 2 compared to those with non-pneumonia infections (Table 2).

**Figure 3:**
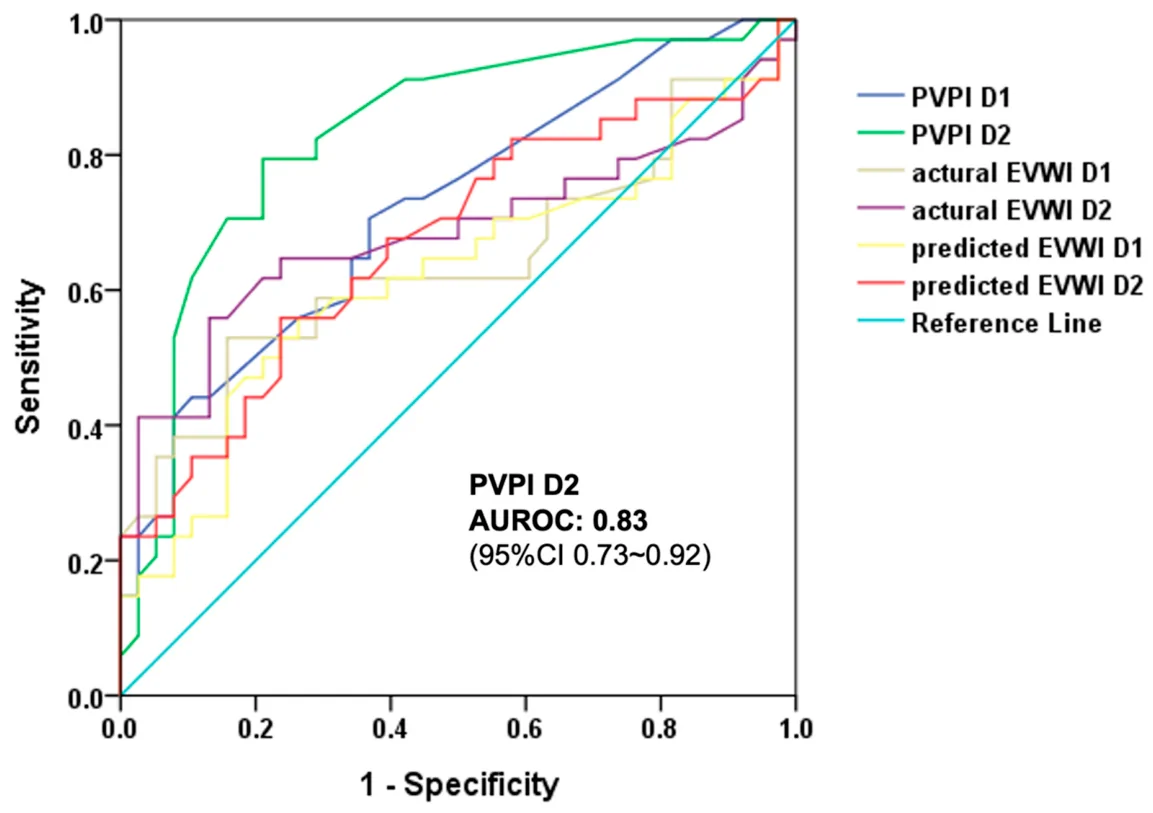
ROC curve of PVPI and EVLWI for predicting 28-day mortality in pneumonia patients.

Within the pneumonia subgroup, 28-day non-survivors exhibited higher PVPI and EVLWI values on both day 1 and day 2 compared to survivors, and the disparities became even more pronounced by day 2, indicating the prognostic values of these PiCCO parameters. The 28-day non-survivors also had significantly lower PaO_2_/FiO_2_ (P/F) ratios than the survivors (Table 3). The subsequent ROC analysis revealed that PVPI D2 was the strongest predictor for 28-day mortality in pneumonia patients among the PiCCO parameters, with an AUROC of 0.83 (95%CI 0.73–0.92) (Figure 3). Using the Youden index, an optimal threshold of PVPI D2 was identified at 2.65. 

Table 4 summarizes the diagnostic performance of PVPI and EVLWI at their respective optimal cut-off values. The bootstrap mean AUC was 0.832 (95% CI: 0.730–0.919), with a mean sensitivity of 79.6% (95% CI: 64.7–92.6%) and a mean specificity of 79.0% (95% CI: 65.6–90.9%), showing minimal optimism compared to the original sample. Across the 1000 bootstrap iterations, the median optimal cut-off determined by the Youden index was 2.70 (mean optimal cut-off: 2.75 ± 0.24; 95% CI: 2.30–3.10), confirming that the chosen cut-off of 2.65 is highly stable within this cohort.

Pneumonia patients with PVPI D2 > 2.65 constituted a high-risk phenotype, exhibiting a nearly four-fold higher 28-day mortality compared to those with PVPI D2 ≤ 2.65 (77.1% vs 18.9%, *p* < 0.001). These high-PVPI patients also had significantly more pronounced extravascular lung water accumulation, higher levels of procalcitonin, and greater disease severity scores compared to the low-PVPI group (Table 5). 

**Figure 4:**
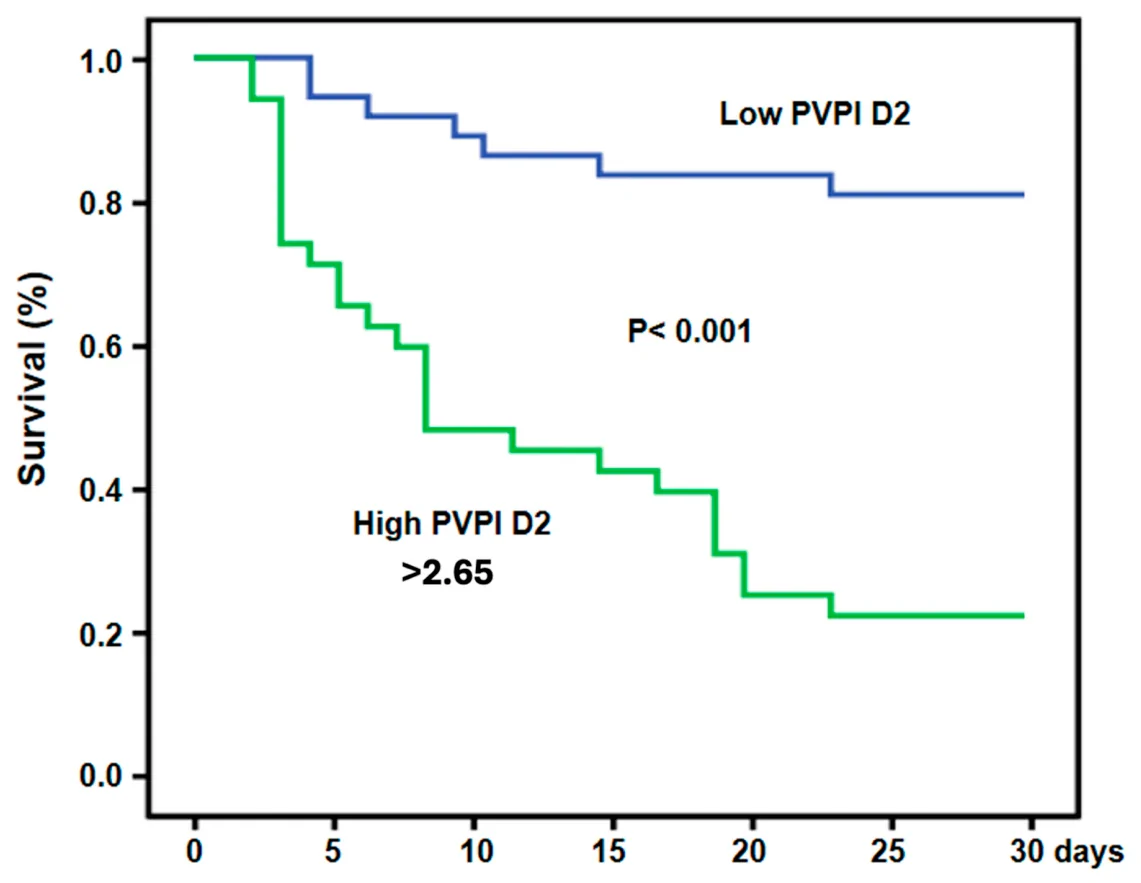
Kaplan–Meier survival analysis in pneumonia patients with high and low PVPI D2.

The Kaplan–Meier analysis further demonstrated that pneumonia patients with PVPI D2 > 2.65 had significantly lower 28-day cumulative survival than those with PVPI D2 ≤ 2.65 (*p* < 0.001) (Figure 4). In a univariate analysis, higher aEVLWI D2, pEVLWI D2, PVPI D2, SOFA D2, APACHE II D2, MELD D2, and lactate D2 values as well as a lower P/F ratio D2 were associated with 28-day mortality in pneumonia patients (Table 6). 

In multivariate analysis, we avoided including variables representing similar physiological constructs simultaneously (e.g., APACHE II and SOFA as global severity scores; EVLWI and PVPI as related pulmonary edema indices) to minimize potential collinearity. We included P/F ratio D2, PVPI D2, lactate D2, SOFA D2, MELD D2, and albumin D2 in the multivariate analysis. PVPI D2 and SOFA D2 were independent risk factors for 28-day mortality (Table 6).

In this multivariable logistic regression analysis (Table 6), with 34 primary endpoint events (28-day mortality) and two predictor variables, the EPV ratio was 17.0, indicating a low risk of overfitting in our model. In addition, higher aEVLWI D2, pEVLWI D2, SOFA D2, APACHE II D2, and lower PaO_2_/FiO_2_ (P/F ratio) D2 values were associated with high pulmonary permeability (PVPI D2 > 2.65) among pneumonia patients in the univariate analysis (Table 7). We included SOFA D2 and P/F ratio D2 in the multivariate analysis to obtain the independent factor associated with high PVPI values. The P/F ratio D2 remained an independent risk factor in the multivariate analysis, supporting an inverse relationship between oxygenation and pulmonary permeability (adjusted OR 0.98, 95%CI 0.98–0.99, *p* = 0.001) (Table 7). PVPI D2 also showed a moderate inverse correlation with the PaO_2_/FiO_2_ ratio (Spearman’s ρ = −0.572, *p* < 0.001). 

Consistently, the P/F ratio D2 showed good discriminative ability for low-permeability status (PVPI D2 ≤ 2.65) in pneumonia patients with an AUROC of 0.80 (95% CI, 0.70–0.90). The ROC curve analysis identified an optimal cut-off value of 163.55 for predicting low permeability status (PVPI on day 2 ≤ 2.65), yielding a sensitivity of 64.9% and a specificity of 85.7%. 

## 4. Discussion

In this cohort of cirrhotic patients with septic shock, we found that patients with non-SBP infections had significantly higher PVPI and EVLWI values than those with SBP. Moreover, pneumonia accounted for the majority of non-SBP infections and was also associated with significantly higher PVPI and EVLWI values. Within the pneumonia subgroup, PVPI on day 2 emerged as an independent predictor of 28-day mortality. Importantly, a lower PaO_2_/FiO_2_ ratio was independently associated with higher pulmonary permeability. 

To our knowledge, this is the first study stratifying cirrhotic patients with septic shock by infection site using PiCCO-derived indices. We identified a pneumonia-specific phenotype characterized by increased PVPI and EVLWI values, which may provide pathophysiological insights into the lack of survival benefit and higher risk of pulmonary edema observed with albumin therapy in this subgroup [5,6,7]. Furthermore, the strong association between elevated PVPI values and 28-day mortality suggests that PVPI may be a useful prognostic marker for risk stratification, potentially reducing patient heterogeneity in future trials of albumin therapy for non-SBP sepsis and guiding fluid resuscitation in cirrhotic patients with septic shock who are at high risk of pulmonary edema. 

The elevation of PVPI and EVLWI values observed in pneumonia-related sepsis in our cohort may reflect the combined effects of direct alveolar−capillary injury [13,14] and the systemic pathophysiological consequences of liver cirrhosis. In cirrhosis, impaired hepatic clearance of endotoxin and cytokines, dysregulated acute-phase protein synthesis, and heightened vasoactive signaling may further amplify pulmonary inflammation [25]. In addition, the liver−lung crosstalk and microbiome shifts can promote oxidative stress in both liver and lung tissues [25]. Taken together, these processes may underlie the distinct pulmonary permeability phenotype we observed in cirrhotic patients with septic shock due to pneumonia. However, these mechanisms were not directly investigated in the present study and should be regarded as hypothesis-generating.

Focusing on the pneumonia subgroup, EVLWI and PVPI values increased from ICU day 1 to day 2 in the 28-day non-survivors, whereas the values of the survivors remained largely stable. A PVPI D2 > 2.65 strongly predicted 28-day mortality and remained significant after adjusting for disease severity by SOFA score. These observations are consistent with prior studies in an acute respiratory distress syndrome (ARDS) patient cohort showing that higher EVLWI and PVPI values were associated with worse outcomes [26] and that early declines in EVLWI within the first 48 h correlated with improved survival [27], likely reflecting an effective reabsorption of extravascular lung water and the attenuation of endothelial injury. 

What distinguishes our study is its infection-site specificity for cirrhotic patients with septic shock. Indeed, our finding of higher PVPI values in patients with pneumonia is consistent with previous ARDS studies which showed greater pulmonary vascular permeability in direct than in indirect lung injury [28]. However, such differences across infection sites have not been well characterized in cirrhosis. This distinction may be particularly relevant given the safety concerns regarding albumin therapy in non-SBP infections, especially the increased risk of pulmonary edema in patients with pneumonia.

Our results extend the literature by showing that the timing and magnitude of PVPI day 2 values are particularly informative when the septic focus is pneumonia. Clinically, these results support early, dynamic risk stratification, whereby a persistently elevated or rising PVPI value in cirrhotic patients with septic shock from pneumonia should prompt a permeability-informed approach, emphasizing restrictive fluid strategies as tolerated. 

Another novel finding of this study is that the P/F ratio may serve as a practical, non-invasive surrogate marker of pulmonary vascular permeability in cirrhotic patients with pneumonia-related septic shock. In this subgroup, a lower P/F ratio was independently associated with higher pulmonary permeability, and P/F ratio also demonstrated good discriminative ability for identifying low-permeability status. 

In our cohort, PVPI D2 was moderately inversely correlated with the PaO_2_/FiO_2_ ratio (Spearman’s ρ = −0.572, *p* < 0.001), supporting the use of the P/F ratio as a practical surrogate marker of pulmonary vascular permeability. These results align with a multicenter ARDS cohort by Kushimoto et al., which showed that increasing ARDS severity, defined by Berlin criteria, was associated with stepwise elevations in EVLWI and PVPI values, and that both parameters were inversely correlated with the P/F ratio (PVPI: r = −0.345, *p* < 0.001) [29]. While the P/F ratio demonstrated good overall discriminative ability (AUROC 0.80), the optimal cut-off showed relatively modest sensitivity (64.9%). Therefore, the P/F ratio should be regarded as a screening marker rather than a substitute for direct PVPI measurement when PiCCO monitoring is available. Taken together, our data support the potential role of the P/F ratio as a non-invasive indicator of pulmonary vascular permeability in cirrhotic patients with septic shock from pneumonia, particularly in settings in which PiCCO monitoring is not available.

Indeed, increased PVPI values in pneumonia may be somewhat expected. Our study is relevant because we quantified its magnitude in cirrhotic patients with septic shock, providing important cut-off values and demonstrated the prognostic value of PVPI, including its association with 28-day mortality and its relationship with the P/F ratio.

Our study has limitations. First, this was a single-center retrospective study, and selection bias cannot be completely excluded. PiCCO was not performed in all eligible patients because of contraindications and rapid clinical improvement. In addition, patients who died on the first day were excluded from the final analysis, which may have introduced survivorship bias. Secondly, our patient population, physician expertise, and local treatment policies may not be representative of other ICU settings. Therefore, our findings should be interpreted with caution and should not be directly extrapolated to other ICU populations, particularly surgical patients or institutions with different case mixes and resuscitation protocols. Third, we were not able to evaluate the impact of albumin infusion on outcomes or PiCCO measurements because the patients in our cohort received albumin as part of the standardized resuscitation strategy for septic shock. Fourth, secondary clinical outcomes such as ICU length of stay, duration of mechanical ventilation, and ventilator-free days were not analyzed. Finally, although the optimal cut-off was internally validated using bootstrap resampling, external validation in independent cohorts is still required before widespread clinical application.

## 5. Conclusions

In cirrhotic patients with septic shock, non-SBP infections, mostly from pneumonia, were associated with elevated PVPI and EVLWI values. Risk stratification according to PVPI in cirrhotic patients with septic shock may help refine fluid resuscitation strategies for patients who are particularly vulnerable to pulmonary edema. Well-designed prospective studies are needed to validate our findings and further elucidate the relationships between PiCCO parameters and multidimensional clinical outcomes in this setting.

## Figures and Tables

**Table 1:** Demographics and measurements of patients with and without SBP.

	All (n = 128)	SBP (n = 15)	Non-SBP (n = 113)	*p* Value
Age (years)	56.7 ± 13.0	60.5 ± 9.5	56.2 ± 13.3	0.230
Male (%)	82	86.7	81.4	1.000
PVPI D1	2.00 (1.90–2.90)	2.00 (1.30–2.00)	2.00 (1.90–3.00)	0.002
aEVLWI D1 (mL/kg)	10.00 (7.93–15.48)	7.00 (6.00–9.00)	10.80 (8.15–16.60)	<0.001
pEVLWI D1 (mL/kg)	11.29 (8.64–18.13)	7.30 (5.00–12.06)	12.00 (9.58–19.06)	0.002
PVPI D2	2.00 (1.93–3.00)	2.00 (1.20–2.00)	2.30 (2.00–3.05)	0.001
aEVLWI D2 (mL/kg)	11.10 (8.00–16.88)	8.00 (6.40–8.30)	11.90 (8.35–17.55)	<0.001
pEVLWI D2 (mL/kg)	12.71 (9.00–19.10)	7.40 (7.00–12.20)	13.40 (10.00–21.51)	<0.001
CRP D1 (mg/L)	63.32 (35.11–110.38)	78.99 (45.24–139.95)	58.13 (32.10–104.87)	0.299
PCT D1 (ng/mL)	3.30 (1.31–15.06)	5.75 (1.89–19.89)	3.10 (1.18–14.93)	0.228
Lactate D1 (mg/dL)	35.20 (19.90–73.45)	38.60 (19.30–127.80)	35.15 (20.00–71.90)	0.506
CRP D2 (mg/L)	70.61 (38.61–116.15)	86.81 (45.24–117.03)	69.70 (36.65–113.12)	0.045
PCT D2 (ng/mL)	5.17 (1.50–15.49)	5.25 (2.55–5.85)	5.15 (1.30–18.20)	0.826
Lactate D2 (mg/dL)	28.3 (16.50–51.40)	25.55 (13.03–64.28)	29.40 (16.70–51.80)	0.604
Albumin D1	2.60 ± 0.52	2.41 ± 0.49	2.62 ± 0.52	0.143
Child-Pugh score D1	11.19 ± 2.13	12.13 ± 2.45	11.06 ± 2.07	0.067
SOFA score D1	14.26 ± 3.17	14.67 ± 3.89	14.21 ± 3.08	0.604
APACHE II score D1	28.14 ± 6.37	27.20 ± 5.00	28.27 ± 6.54	0.542
MELD score D1	29.38 ± 8.55	30.33 ± 11.17	29.25 ± 8.20	0.646
Albumin D2	2.71 ± 0.43	2.73 ± 0.38	2.71 ± 0.44	0.838
Child-Pugh score D2	11.16 ± 2.16	11.67 ± 2.72	11.09 ± 2.08	0.335
SOFA score D2	14.99 ± 3.56	14.27 ± 3.79	15.09 ± 3.54	0.403
APACHE II score D2	28.13 ± 7.67	26.27 ± 4.64	28.38 ± 7.97	0.317
MELD score D2	30.18 ± 9.10	31.07 ± 10.75	30.06 ± 8.91	0.689
28-day mortality (%)	46.4	53.3	44.2	0.507

Abbreviations: D1, day 1; D2, day 2; EVLWI, extravascular lung water index; aEVLWI, EVLW indexed to actual body weight; pEVLWI, EVLW indexed to predicted body weight; PVPI, pulmonary vascular permeability index; SOFA, Sequential Organ Failure Assessment; MELD, Model for End-Stage Liver Disease; APACHE II, Acute Physiology and Chronic Health Evaluation II; CRP, C-reactive protein; PCT, procalcitonin.

**Table 2:** Demographics and measurements of patients with and without pneumonia.

	All (n = 128)	Pneumonia (n = 72)	Non-Pneumonia (n = 56)	*p* Value
Age (years)	56.7 ± 13.0	56.4 ± 13.6	57.2 ± 12.2	0.739
Male (%)	82	80.6	83.9	0.622
PVPI D1	2.00 (1.90–2.90)	2.65 (2.00–3.20)	2.00 (1.53–2.00)	<0.001
aEVLWI D1 (mL/kg)	10.00 (7.93–15.48)	14.30 (10.00–19.00)	8.10 (6.70–9.50)	<0.001
pEVLWI D1 (mL/kg)	11.29 (8.64–18.13)	15.78 (11.07–21.96)	9.00 (6.41–11.00)	<0.001
PVPI D2	2.00 (1.93–3.00)	2.60 (2.00–3.83)	2.00 (1.50–2.00)	<0.001
aEVLWI D2 (mL/kg)	11.10 (8.00–16.88)	15.00 (10.28–21.60)	8.00 (7.00–11.08)	<0.001
pEVLWI D2 (mL/kg)	12.71 (9.00–19.10)	17.30 (12.44–24.93)	9.29 (7.03–12.08)	<0.001
CRP D1 (mg/L)	63.32 (35.11–110.38)	58.13 (38.72–110.24)	69.35 (24.90–122.47)	0.756
PCT D1 (ng/mL)	3.30 (1.31–15.06)	2.83 (1.06–9.14)	5.66 (1.56–20.69)	0.029
Lactate D1 (mg/dL)	35.20 (19.90–73.45)	26.30 (17.30–60.65)	46.30 (24.80–89.80)	0.041
CRP D2 (mg/L)	70.61 (38.61–116.15)	70.65 (37.83–115.79)	68.10 (38.77–116.90)	0.619
PCT D2 (ng/mL)	5.17 (1.50–15.49)	3.04 (1.40–11.60)	5.96 (1.48–22.39)	0.156
Lactate D2 (mg/dL)	28.3 (16.50–51.40)	26.60 (16.70–49.70)	33.15 (15.68–63.20)	0.567
Albumin D1	2.60 ± 0.52	2.64 ± 0.48	2.54 ± 0.56	0.315
Child-Pugh score D1	11.19 ± 2.13	11.07 ± 2.02	11.34 ± 2.28	0.48
SOFA score D1	14.27 ± 3.17	14.63 ± 2.88	13.80 ± 3.48	0.146
APACHE II score D1	28.15 ± 6.37	29.82 ± 6.41	26.00 ± 5.70	0.001
MELD score D1	29.37 ± 8.55	28.32 ± 8.09	30.73 ± 9.01	0.114
Albumin D2	2.71 ± 0.43	2.72 ± 0.46	2.70 ± 0.40	0.716
Child-Pugh score D2	11.16 ± 2.16	11.13 ± 2.09	11.20 ± 2.27	0.851
SOFA score D2	14.99 ± 3.57	15.61 ± 3.25	14.21 ± 3.82	0.028
APACHE II score D2	28.14 ± 7.67	30.54 ± 7.26	25.09 ± 7.12	<0.001
MELD score D2	30.18 ± 9.10	29.28 ± 8.23	31.34 ± 10.06	0.205
28-day mortality (%)	45.3	47.2	42.9	0.623

Abbreviations: D1, day 1; D2, day 2; EVLWI, extravascular lung water index; aEVLWI, EVLW indexed to actual body weight; pEVLWI, EVLW indexed to predicted body weight; PVPI, pulmonary vascular permeability index; SOFA, Sequential Organ Failure Assessment; MELD, Model for End-Stage Liver Disease; APACHE II, Acute Physiology and Chronic Health Evaluation II; CRP, C-reactive protein; PCT, procalcitonin.

**Table 3:** Demographics and measurements of pneumonia patients stratified by 28-day survival.

	All (n = 72)	Pneumonia 28D Death (n = 34)	Pneumonia 28D Survive (n = 38)	*p* Value
Age (years)	56.4 ± 13.6	55.4 ± 13.9	57.3 ± 13.6	0.552
Male (%)	80.6	82.4	78.9	0.715
PVPI D1	2.65 (2.00–3.20)	3.00 (2.08–5.03)	2.05 (1.90–3.00)	0.001
aEVLWI D1 (mL/kg)	14.30 (10.00–19.00)	17.30 (9.78–26.50)	13.75 (10.00–16.30)	0.037
pEVLWI D1 (mL/kg)	15.78 (11.07–21.96)	19.61 (10.95–24.39)	13.85 (11.21–19.05)	0.075
PVPI D2	2.60 (2.00–3.83)	3.30 (2.70–5.15)	2.00 (1.95–2.60)	<0.001
aEVLWI D2 (mL/kg)	15.00 (10.28–21.60)	19.95 (11.03–29.08)	13.55 (10.08–16.85)	0.007
pEVLWI D2 (mL/kg)	17.30 (12.44–24.93)	21.81 (14.77–29.50)	15.30 (11.39–21.50)	0.012
CRP D1 (mg/L)	58.13 (38.72–110.24)	68.63 (39.16–112.42)	56.69 (36.58–92.80)	0.621
PCT D1 (ng/mL)	2.83 (1.06–9.14)	2.73 (1.06–5.18)	3.94 (1.00–14.07)	0.523
Lactate D1 (mg/dL)	26.30 (17.30–60.65)	42.50 (23.28–90.93)	21.20 (13.60–38.80)	<0.001
PaO_2_/FiO_2_ ratio D1	144.00 (101.28–231.80)	118.80 (79.33–176.48)	190.30 (128.13–278.45)	0.002
CRP D2 (mg/L)	70.65 (37.83–115.79)	81.42 (41.02–118.09)	67.85 (35.94–115.79)	0.630
PCT D2 (ng/mL)	3.04 (1.40–11.60)	2.86 (1.55–6.12)	5.26 (1.27–13.26)	0.462
Lactate D2 (mg/dL)	26.60 (16.70–49.70)	32.80 (22.75–105.00)	24.45 (10.23–33.95)	0.010
PaO_2_/FiO_2_ ratio D2	145.50 (96.95–203.93)	101.70 (72.35–149.83)	177.85 (141.83–268.98)	<0.001
Albumin D1	2.64 ± 0.48	2.52 ± 0.55	2.75 ± 0.39	0.042
Child-Pugh score D1	11.07 ± 2.02	11.35 ± 1.84	10.82 ± 2.15	0.262
SOFA score D1	14.63 ± 2.88	15.68 ± 2.32	13.68 ± 3.03	0.003
APACHE II score D1	29.82 ± 6.41	31.38 ± 5.37	28.42 ± 6.99	0.05
MELD score D1	28.32 ± 8.09	29.41 ± 7.62	27.34 ± 8.48	0.282
Albumin D2	2.72 ± 0.46	2.63 ± 0.53	2.81 ± 0.37	0.091
Child-Pugh score D2	11.13 ± 2.09	11.50 ± 1.75	10.78 ± 2.34	0.151
SOFA score D2	15.61 ± 3.25	17.18 ± 2.84	14.16 ± 2.93	<0.001
APACHE II score D2	30.54 ± 7.26	33.38 ± 7.27	27.92 ± 6.27	0.001
MELD score D2	29.28 ± 8.23	31.74 ± 8.15	27.08 ± 7.76	0.016
28-day mortality (%)	47.2	100.0	0.0	—

Abbreviations: D1, day 1; D2, day 2; EVLWI, extravascular lung water index; aEVLWI, EVLW indexed to actual body weight; pEVLWI, EVLW indexed to predicted body weight; PVPI, pulmonary vascular permeability index; SOFA, Sequential Organ Failure Assessment; MELD, Model for End-Stage Liver Disease; APACHE II, Acute Physiology and Chronic Health Evaluation II; CRP, C-reactive protein; PCT, procalcitonin.

**Table 4:** Diagnostic performance of PVPI and EVLWI at their respective optimal cut-off values in patients with pneumonia.

	AUROC	Cut of Point	Sensitivity	Specificity	PPV	NPV	Accuracy
PVPI D1	0.720 (0.602–0.837)	3.15	44.1%	89.5%	78.9%	64.2%	68.1%
PVPI D2	0.830 (0.732–0.929)	2.65	79.4%	78.9%	77.1%	81.1%	79.2%
aEVLWI D1	0.643 (0.509–0.777)	17.25	44.1%	89.5%	78.9%	64.2%	68.1%
aEVLWI D2	0.685 (0.552–0.817)	19.05	55.9%	86.8%	79.2%	68.8%	72.2%
pEVLWI D1	0.622 (0.488–0.756)	18.23	55.9%	73.7%	65.5%	65.1%	65.3%
pEVLWI D2	0.671 (0.544–0.799)	21.11	55.9%	76.3%	67.9%	65.9%	66.7%

Abbreviations: D1, day 1; D2, day 2; EVLWI, extravascular lung water index; aEVLWI, EVLW indexed to actual body weight; pEVLWI, EVLW indexed to predicted body weight; PVPI, pulmonary vascular permeability index; PPV, positive predictive value; NPV, negative predictive value.

**Table 5:** Demographics and measurements of pneumonia patients stratified by PVPI D2.

	All (n = 72)	Pneumonia High PVPI D2(>2.65) (n = 35)	Pneumonia Low PVPI D2(<2.65) (n = 37)	*p* Value
Age (years)	56.4 ± 13.6	57.1 ± 14.3	55.8 ± 13.2	0.695
Male (%)	80.6	74.3	86.5	0.191
PVPI D1	2.65 (2.00–3.20)	3.20 (2.80–5.10)	2.00 (1.90–2.30)	<0.001
aEVLWI D1 (mL/kg)	14.30 (10.00–19.00)	18.90 (15.00–25.40)	10.90 (8.35–14.30)	<0.001
pEVLWI D1 (mL/kg)	15.78 (11.07–21.96)	21.43 (15.70–25.00)	12.00 (10.09–16.21)	<0.001
PVPI D2	2.60 (2.00–3.83)	3.90 (3.00–6.00)	2.00 (1.80–2.30)	<0.001
aEVLWI D2 (mL/kg)	15.00 (10.28–21.60)	21.30 (16.90–29.40)	11.30 (9.00–14.95)	<0.001
pEVLWI D2 (mL/kg)	17.30 (12.44–24.93)	24.00 (17.95–28.70)	13.00 (10.41–16.77)	<0.001
CRP D1 (mg/L)	58.13 (38.72–110.24)	70.59 (46.12–110.43)	50.71 (30.30–106.11)	0.290
PCT D1 (ng/mL)	2.83 (1.06–9.14)	1.61 (0.95–4.43)	5.83 (1.45–16.02)	0.008
Lactate D1 (mg/dL)	26.30 (17.30–60.65)	26.15 (21.05–61.03)	26.30 (15.95–61.25)	0.727
PaO_2_/FiO_2_ ratio D1	144.00 (101.28–231.80)	121.00 (80.30–169.00)	220.40 (129.55–317.30)	<0.001
CRP D2 (mg/L)	70.65 (37.83–115.79)	84.20 (43.48–121.08)	62.01 (34.75–109.26)	0.381
PCT D2 (ng/mL)	3.04 (1.40–11.60)	1.88 (1.07–5.21)	7.00 (2.36–17.83)	0.008
Lactate D2 (mg/dL)	26.60 (16.70–49.70)	25.80 (21.40–52.45)	28.30 (14.45–45.70)	0.727
PaO_2_/FiO_2_ ratio D2	145.50 (96.95–203.93)	113.80 (72.40–146.60)	194.00 (135.55–286.25)	<0.001
Albumin D1	2.64 ± 0.48	2.58 ± 0.53	2.69 ± 0.44	0.320
Child-Pugh score D1	11.07 ± 2.02	10.83 ± 2.18	11.30 ± 1.85	0.328
SOFA score D1	14.63 ± 2.88	14.97 ± 2.93	14.30 ± 2.84	0.324
APACHE II score D1	29.82 ± 6.41	30.31 ± 6.27	29.35 ± 6.58	0.528
MELD score D1	28.32 ± 8.09	26.91 ± 8.40	29.65 ± 7.67	0.153
Albumin D2	2.72 ± 0.46	2.67 ± 0.51	2.78 ± 0.41	0.341
Child-Pugh score D2	11.13 ± 2.09	11.00 ± 2.00	11.25 ± 2.20	0.618
SOFA score D2	15.61 ± 3.25	16.46 ± 3.22	14.78 ± 3.09	0.028
APACHE II score D2	30.54 ± 7.26	32.80 ± 7.30	28.33 ± 6.60	0.009
MELD score D2	29.28 ± 8.23	28.34 ± 8.94	30.16 ± 7.52	0.352
28-day mortality (%)	47.2	77.1	18.9	<0.001

Abbreviations: D1, day 1; D2, day 2; EVLWI, extravascular lung water index; aEVLWI, EVLW indexed to actual body weight; pEVLWI, EVLW indexed to predicted body weight; PVPI, pulmonary vascular permeability index; SOFA, Sequential Organ Failure Assessment; MELD, Model for End-Stage Liver Disease; APACHE II, Acute Physiology and Chronic Health Evaluation II; CRP, C-reactive protein; PCT, procalcitonin.

**Table 6:** Univariate and multivariate analyses to predict 28-day outcomes in patients with pneumonia.

Parameter	Beta Coefficient	Standard Error	Odds Ratios (95%CI)	*p*
***Univariate logistic regression***				
Age (years)	−0.011	0.018	0.989 (0.956–1.024)	0.546
Sex (M/F)	0.219	0.601	1.244 (0.384–4.038)	0.716
aEVLWI D2 (mL/kg)	0.112	0.036	1.118 (1.041–1.201)	0.002
pEVLWI D2 (mL/kg)	0.082	0.031	1.086 (1.021–1.155)	0.009
PVPI D2	0.782	0.236	2.186 (1.375–3.475)	0.001
Albumin D2	−0.925	0.554	0.397 (0.134–1.176)	0.095
Child–Pugh score D2	0.172	0.120	1.187 (0.939–1.502)	0.152
SOFA score D2	0.376	0.107	1.457 (1.181–1.797)	<0.001
APACHE II D2	0.123	0.041	1.130 (1.042–1.226)	0.003
MELD D2	0.075	0.032	1.078 (1.012–1.148)	0.020
CRP D2 (mg/L)	0.001	0.004	1.001 (0.994–1.009)	0.771
PCT D2 (ng/mL)	0.002	0.011	1.002 (0.981–1.024)	0.845
Lactate D2 (mg/dL)	0.016	0.008	1.016 (1.001–1.031)	0.035
PaO_2_/FiO_2_ ratio D2	−0.009	0.003	0.991 (984–0.997)	0.006
***Multivariate logistic regression***				
PVPI D2	0.615	0.239	1.850 (1.157–2.958)	0.010
SOFA score D2	0.368	0.131	1.445 (1.119–1.867)	0.005

Abbreviations: EVLWI, extravascular lung water index; PVPI, pulmonary vascular permeability index; aEVLWI, EVLW indexed to actual body weight; pEVLWI, EVLW indexed to predicted body weight; SOFA, Sequential Organ Failure Assessment; APACHE II, Acute Physiology and Chronic Health Evaluation II; MELD, Model for End-Stage Liver Disease; MAP, mean arterial pressure; CRP, C-reactive protein; PCT, procalcitonin; D2, day 2.

**Table 7:** Univariate and multivariate analyses to predict high PVPI D2 (>2.65) values in patients with pneumonia.

Parameter	Beta Coefficient	Standard Error	Odds Ratios (95%CI)	*p*
***Univariate logistic regression***				
Age (years)	0.007	0.017	1.007 (0.973–1.042)	0.690
Sex (M/F)	−0.795	0.617	0.451 (0.135–1.513)	0.197
aEVLWI D2 (mL/kg)	0.314	0.075	1.369 (1.181–1.586)	<0.001
pEVLWI D2 (mL/kg)	0.205	0.049	1.228 (1.116–1.351)	<0.001
Albumin D2	−0.507	0.529	0.602 (0.214–1.697)	0.337
Child−Pugh score D2	−0.058	0.115	0.943 (0.753–1.182)	0.612
SOFA score D2	0.174	0.082	1.189 (1.014–1.396)	0.034
APACHE II D2	0.095	0.038	1.100 (1.020–1.185)	0.013
MELD D2	−0.028	0.029	0.973 (0.919–1.030)	0.348
CRP D2 (mg/L)	0.003	0.004	1.003 (0.995–1.011)	0.457
PCT D2 (ng/mL)	−0.020	0.013	0.981 (0.956–1.006)	0.137
Lactate D2 (mg/dL)	0.003	0.004	1.003 (0.995–1.011)	0.403
PaO_2_/FiO_2_ ratio D2	−0.017	0.005	0.983 (0.974–0.993)	<0.001
***Multivariate logistic regression***				
PaO_2_/FiO_2_ ratio D2	−0.016	0.005	0.984 (0.975–0.993)	0.001

Abbreviations: EVLWI, extravascular lung water index; PVPI, pulmonary vascular permeability index; aEVLWI, EVLW indexed to actual body weight; pEVLWI, EVLW indexed to predicted body weight; SOFA, Sequential Organ Failure Assessment; APACHE II, Acute Physiology and Chronic Health Evaluation II; MELD, Model for End-Stage Liver Disease; MAP, mean arterial pressure; CRP, C-reactive protein; PCT, procalcitonin; D2, day 2.

## Data Availability

The datasets used and/or analyzed during the current study are available from the corresponding author on reasonable request.

## Abbreviations

EVLWI, extravascular lung water index; PVPI, pulmonary vascular permeability index; aEVLWI, EVLW indexed to actual body weight; pEVLWI, EVLW indexed to predicted body weight; SOFA, Sequential Organ Failure Assessment; APACHE II, Acute Physiology and Chronic Health Evaluation II; MELD, Model for End-Stage Liver Disease; CRP, C-reactive protein; PCT, procalcitonin.
